# Dissecting the Genetics of Early Vigour to Design Drought-Adapted Wheat

**DOI:** 10.3389/fpls.2021.754439

**Published:** 2022-01-03

**Authors:** Stjepan Vukasovic, Samir Alahmad, Jack Christopher, Rod J. Snowdon, Andreas Stahl, Lee T. Hickey

**Affiliations:** ^1^Department of Plant Breeding, IFZ Research Centre for Biosystems, Land Use and Nutrition, Justus Liebig University Giessen, Giessen, Germany; ^2^Queensland Alliance for Agriculture and Food Innovation, The University of Queensland, Brisbane, QLD, Australia; ^3^Leslie Research Facility, Queensland Alliance for Agriculture and Food Innovation, The University of Queensland, Toowoomba, QLD, Australia; ^4^Federal Research Centre for Cultivated Plants, Institute for Resistance Research and Stress Tolerance, Julius Kühn-Institute, Quedlinburg, Germany

**Keywords:** *Triticum aestivum*, normalised difference vegetation index, NDVI, nested association mapping, genome-wide association study, GWAS

## Abstract

Due to the climate change and an increased frequency of drought, it is of enormous importance to identify and to develop traits that result in adaptation and in improvement of crop yield stability in drought-prone regions with low rainfall. Early vigour, defined as the rapid development of leaf area in early developmental stages, is reported to contribute to stronger plant vitality, which, in turn, can enhance resilience to erratic drought periods. Furthermore, early vigour improves weed competitiveness and nutrient uptake. Here, two sets of a multi-reference nested association mapping (MR-NAM) population of bread wheat (*Triticum aestivum* ssp. *aestivum* L.) were used to investigate early vigour in a rain-fed field environment for 3 years, and additionally assessed under controlled conditions in a greenhouse experiment. The normalised difference vegetation index (NDVI) calculated from red/infrared light reflectance was used to quantify early vigour in the field, revealing a correlation (*p* < 0.05; *r* = 0.39) between the spectral measurement and the length of the second leaf. Under controlled environmental conditions, the measured projected leaf area, using a green-pixel counter, was also correlated to the leaf area of the second leaf (*p* < 0.05; *r* = 0.38), as well as to the recorded biomass (*p* < 0.01; *r* = 0.71). Subsequently, genetic determination of early vigour was tested by conducting a genome-wide association study (GWAS) for the proxy traits, revealing 42 markers associated with vegetation index and two markers associated with projected leaf area. There are several quantitative trait loci that are collocated with loci for plant developmental traits including plant height on chromosome 2D (log_10_ (*P*) = 3.19; PVE = 0.035), coleoptile length on chromosome 1B (–log_10_ (*P*) = 3.24; PVE = 0.112), as well as stay-green and vernalisation on chromosome 5A (–log_10_ (*P*) = 3.14; PVE = 0.115).

## Introduction

Global climate change is considered one of the biggest and most complex challenges the mankind has faced. One effect that has been observed since 1970, which leads to severe yield losses, is the increased occurrence of erratic drought phenomena (Liu et al., 2019). Specifically, the agricultural sector is facing serious challenges since drought-stress is considered the most limiting factor in rain-fed cropping systems (Hu and Xiong, 2014). Based on calculations of the Intergovernmental Panel on Climate Change (IPCC), it is predicted that the global mean surface temperature will rise 2°C more in the 20-year period from 2046 to 2065, than in the comparable period between 1986 and 2005, with a total increase of 4.8°C by 2100 (IPCC, 2015). As a result, more frequent heat and drought events are to be expected and to be classified as a major threat to the primary production sector in general and the wheat production in particular (Steinfort et al., 2017). Based on crop modelling scenarios, it is predicted that global wheat production will fall by 6% per 1°C temperature increase (Asseng et al., 2013). In particular, the Australian wheat production region is expected to experience a strong increase in drought and heat events, with a yield decrease of up to 20% projected from a median temperature increase of 2°C (Asseng et al., 2015). The severe impact of strong drought events has already been observed during the “Millennium Drought” between 2001 and 2009, where major reductions in production were recorded. In southern Australia in particular, production was severely decreased due to the impact of drought during this phase (Dijk et al., 2013). Large areas of the southern and western wheat-cropping regions in Australia have a Mediterranean climate, which is defined by terminal droughts (Siddique et al., 1990; Whan et al., 1991; Botwright et al., 2002; Rebetzke et al., 2008; Sadras and Dreccer, 2015; Rebetzke et al., 2017).

Consequently, there has been a growing focus on commercial and public breeding programs to identify traits associated with water-use efficiency to increase the yield potential under water-limited conditions (Lopes and Reynolds, 2012; Passioura, 2012). Potential traits of interest include long coleoptiles, which enable deeper sowing in the soil profile and improved access to water reservoirs underneath a dry surface soil, as well as reduced tillering to lessen unnecessarily metabolism into the non-fertile emerging tillers (Richards et al., 2010). Another is early vigour (EV), defined as the rapid production of leaf area during the early development phase of the plant (López-Castañeda et al., 1996). The primary advantage of EV is the increased biomass production early in the season and the rapid closure of the canopy, which can reduce evaporation of soil water, which then increases water availability (López-Castañeda and Richards, 1994; Condon et al., 2004). Early canopy closure also leads to minimized solar radiation on the soil and to an enhanced competitiveness of the crop against weeds (Dingkuhn et al., 1999; Coleman et al., 2001; Lemerle et al., 2001; Bertholsson, 2005). Mediterranean growing areas are water-limited environments which are characterized by experiencing late seasons droughts. According to Richards et al. (1987) and López-Castañeda et al. (1995), EV offers great potential for increasing water-use efficiency in such drought-prone regions. Additional advantages that may be associated with EV include larger uptake of essential plant nutrients, superior tolerance to aluminium stress, as well as improved yield under high temperatures and elevated atmospheric CO_2_ concentration (Coleman et al., 2001; Lemerle et al., 2001; Liao et al., 2004; Bertholsson, 2005; Ludwig and Asseng, 2010; Valle and Calderini, 2010; Ryan et al., 2015). Previous studies in wheat have already identified various physiological traits associated with EV, such as the embryo size (Moore and Rebetzke, 2015), coleoptile length (Clarke et al., 1991; Rebetzke et al., 2007), tiller size, and leaf characteristics (Rebetzke and Richards, 1999; Rebetzke et al., 2007, 2017).

Several studies have highlighted the beneficial effect of increased EV on yield performance in specific environments. Nevertheless, due to insufficient knowledge about genetic variation and lack of information on the economic value of the trait, EV has only been introduced into breeding programs to a limited degree (Rebetzke et al., 2017). However, Botwright et al. (2002) demonstrated the positive effect of EV on yield performance in medium and low rainfall regions in combination with favourable soil conditions, such as sandy soils. Early vigour has significant implications for water demand. For example, a slight increase in leaf area growth during the vegetative growth stages can lead to an increase in biomass, transpiration area, and water use (Asseng and van Herwaarden, 2003). This can result in rapid depletion of soil water prior to anthesis, which may have a negative impact on grain yield during flowering time and grain filling (Richards and Townley-Smith, 1987). According to Turner and Nicolas (1998), strong vigorous genotypes have deeper and greater water uptake compared to less vigorous genotypes and are subsequently considered to be advantageous in low rainfall environments, including Mediterranean growing regions. However, despite a clear genetic effect on EV, its interaction with the environment, soil type, and available fertilizer is substantial. For example, EV may adversely affect yield if managed unfavourably. Therefore, ensuring that the crop is provided with sufficient nitrogen (N) is essential in order to prevent premature N deficiency due to excessive biomass production (Asseng and van Herwaarden, 2003).

To determine the impact of EV on yield, a better understanding of the physiological mechanisms of the trait along with its genetic control is required. In addition, efficient and low-cost phenotyping procedures are needed to assess EV in wheat. The aim of this study was (i) to investigate the physiological characteristics that drive EV in wheat in the field and under controlled conditions, (ii) to assess the efficiency of phenotyping methodologies under greenhouse and field conditions, and (iii) to identify genomic regions influencing EV in wheat.

## Materials and Methods

### Plant Material

To evaluate EV in the field, a set of 685 spring wheat genotypes (further referred to as Set 1) was randomly selected from a multi-reference nested association mapping population (MR-NAM). The MR-NAM population was developed based on 11 diverse founder lines which were crossed with the commercially used wheat varieties Suntop (AGT), Scout (LPB), and Mace (AGT), then consequently adapted to the environmental conditions of the western, northern, and southern cropping regions of Australia, respectively (Richard et al., 2015). The founder lines were selected according to key traits, such as drought adaptation and stay-green (e.g., Dharwar Dry, Drysdale), root architecture traits (e.g., Seri) or adaptation to nematodes, and disease resistance (e.g., Wylie, Gregory) (Richard et al., 2015; Christopher et al., 2021). After crossing the founder lines with the three parental lines, using an incomplete crossing scheme, the 15 F_1_ lines were generated. Subsequently, these 15 F_1_ lines were used for population development through inbreeding, which produces1474 F_4_-derived lines and were then segmented into 15 genetically diverse families. These 15 families comprised four Mace-derived families, forming a conventional NAM population which was denoted as the Mace-NAM (Ma-NAM) component of the MR-NAM, five Scout-derived families (Sc-NAM), and six Suntop-derived families (Su-NAM). The NAM population was genotyped using the DArT-seq genotype-by-sequencing platform, producing over 25,000 polymorphic markers (Richard et al., 2015). The first set (SET 1) was tested in experimental years, 2015 and 2016, respectively. In 2017, a randomly selected subset comprising 210 lines (referred to as Set 2) was selected and was tested in the field in a greenhouse (GH) environment. In order to provide a more detailed information of the physiological characteristics, a core set was formed within Set 2, which was intensively investigated in the field and in the GH experiment.

### Experimental Design

#### Evaluating Early Vigour Under Field Conditions

Field trials were conducted over 3 years from 2015 to 2017. All field trials were carried out under rain-fed conditions at the Hermitage Research Facility (HRF), Warwick, Queensland, Australia (28.21°S, 152.10°E, 480 m above sea level). The HRF site is characterized by alkaline, cracking, and heavy clay soils with high water-holding capacity. Cropping season is from May to October, with an average rainfall of 211 mm and an average temperature of 14°C. Further information regarding the environmental conditions is given in Table 1. Sites were sown with yield plots, each plot measuring 2 m × 6 m, containing 7 rows at 25 cm spacing, with a target crop density of 100 plants/m^2^. To precisely reach the target crop density, thousand seed weight and germination rate were determined for seed of each genotype, while the sowing rates were calculated for each genotype. To avoid any artefacts in seed size, the seeds of the lines used in each trial were sourced from a common site and the year of the seed propagation. Seeds were generated from fully irrigated seed-increase rows sown at 0.5 m row spacing and fertilizer was applied to provide for the non-limiting conditions for both water and nutrients. Furthermore, diseases and weeds were controlled as necessary. This allowed for the full potential seed size of each genotype to be expressed during seed production. In all trials, no specific selection for seed size were performed. In each year the trials received 120 kg/ha^–1^ of urea prior to sowing, and 40 kg/ha^–1^ of Starter Z^®^ (Incitec Pivot Fertilisers, Southbank, VIC, Australia; 10.5% N, 19.5% P, 2.2% S, 2.2% Zn) was applied at sowing. Plant protection measures were applied as necessary. All field trials were designed as a partially replicated (p-rep) block design with percentage of partial replication of 34, 29, and 62% in 2015, 2016, and 2017, respectively.

**TABLE 1:** Details for environmental conditions for wheat trials subjected to analyses in this study.

Trial	Location	State	Sowing date	CIR [mm]	PAWC [mm]	AvgT [°C]	RAD (MJ m^–2^)
NAM 2015	HRF, Warwick	QLD	11.06.2015	103.4	329.6	13.1	2,318
NAM 2016	HRF, Warwick	QLD	22.07.2016	303.1	212.1	14.9	2,430
NAM 2017	HRF, Warwick	QLD	27.05.2017	112.4	286.47	14.2	2,276

*Shon are trials name and year (Trial), location and state, sowing date, cumulative in-crop rainfall in mm (CIR), plant available water capacity of the soil in mm (PAWC), daily average temperature from sowing to maturity in °C (AvgT), and cumulative radiation from sowing to maturity in MJ m^–2^ (RAD).*

Across the field trials, the normalised difference vegetation index (NDVI) was used as a quantitative measure for EV. The NDVI measurements were collected using a hand-held NTech Greenseeker^®^ model 505, manufactured by NTech Industries, Ukiah, CA, United States. By attaching the device to the body by using a harness, the measurement could be carried out constantly at a height of 1 m. The NDVI has been reported as a very useful index for studying the dynamics of canopy development and of senescence patterns of wheat (Lopes and Reynolds, 2012; Christopher et al., 2014). This vegetation index has also been previously used to evaluate EV in wheat (Li et al., 2014). In this study, NDVI measurements were collected at 29 days after sowing (DAS) for all field trials.

In 2017, detailed measures were captured to fully understand the physiological properties. A core set consisting of 30 genotypes was compiled from Set 2 and was intensively examined at the time of the NDVI measurement. The core set included all founder and reference lines from the MR-NAM population, along with selected good-performing lines from other unpublished experiments. From each of the 30 genotypes, ten plants per plot were randomly sampled for each genotype, while the leaf characteristics were recorded by measuring the length and width of the first (L1) and the second leaf (L2). Subsequently, the approximate leaf area for L1 and L2 was calculated by multiplying the measured length by the width. The sum of the calculated leaf areas of L1 and L2 was then expressed as the total leaf area (TLA).

#### Evaluating Early Vigour Under Controlled Conditions

The GH experiment was conducted using 210 genotypes from Set 2. The greenhouse chamber temperature was set on 22°C during daytime and 17°C during night time, while the lighting conditions were set to provide a 12-h photoperiod. Furthermore, the plants were irrigated on a daily basis. A single plant was grown per pot using 250 ml pots with 70 mm diameter. The potting media had a pH of 5.5–6.5 and was a composition of 70% pine bark (0–5 mm) and 30% coco peat, as well as fertilizers. Furthermore, Osmocote^®^ (ICL SF, Sydney, NSW, Australia), containing 19.4% N, 16% P, and 5% K, was added to the potting media to guarantee sufficient nutrients during the experiment. The trial was designed as a fully replicated, randomized, and complete block design with six replications per genotype.

**FIGURE 1:**
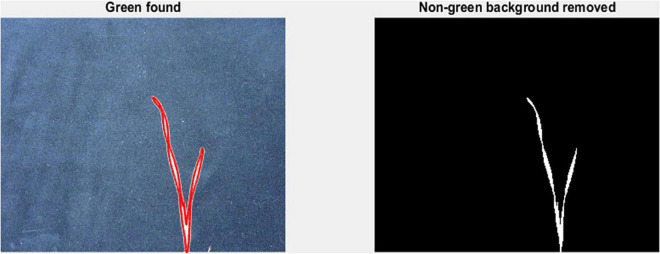
Mode of operation of the green pixel counter, analysis takes place in two steps. First capture of the green pixels in the image as indicated by the red outlined area (left). Second, removal of all non-green pixels (right).

To measure leaf dimensions on a larger scale within the GH experiment setup, this study evaluated the extent of the image analysis methods that will be suitable for this purpose. For this purpose, a green pixel counter was programmed using the MATLAB^®^ (MathWorks, Inc., Natick, MA, United States) programming language. The pixel counter identifies green pixels in an image to calculate a projected leaf area (PLA) and is a low-cost approach for image analysis (Figure 1). The measured PLA, using the green pixel counter, is analogous to the TLA, estimated as the sum of the areas of L1 and L2, as calculated from the length and width of each. With the default settings, images were taken using a Canon Eos 750D^®^ camera. To reduce variation between images, the camera was mounted on a tripod at 80 cm distance from a platform on which every pot was placed. To avoid interference with other pixels or light sources, the image was taken in a closed room with consistent lighting conditions. In addition, a black background was placed behind the plant to exclude any other colour pigments from the picture, reducing them only to black and green pigments. The image analysis was conducted two times: first at 17 DAS and second at 21 DAS. At 17 DAS, images were captured for genotypes in Set 2. At 21 DAS all replications of the core set of Set 2 were imaged, as well as recorded manually. Again, the length and width of L1 and L2 were measured.

### Statistical Analyses of Phenotype Data

For the calculation of the best linear unbiased estimators (BLUEs), the linear mixed model (LMM) described in Eq. 1 was used. For the calculation of the LMM, the R-language-based packages lme4^1^ combined with lsmeans^2^ were used.


(1) $$P_{i\,j\,k\,l}=\mu +g_{i}+W_{k}+C_{j}+R_{l}+e_{i\,j\,k\,l}$$

where *P*_*ijkl*_ is the phenotypic value of the *i^th^* genotype, in the *k^th^* replication, μ stands for the overall mean, *g*_*i*_ describes the fixed effects of the *i^th^* genotype. The random effects are *W*_*k*_, which is the *k^th^* replication, *C*_*j*_, which represents the *j^th^* column, and *R*_*l*_ representing the *l^th^* row. The error term is represented by *e*_*ijkl*_.

Pearson correlation matrices were created using the R package psych. Principal component analysis was conducted using the R package stats. For the Pearson correlation, as well as for the principal component analysis, we used the BLUEs described in Eq. 1.

### Association Mapping

Association mapping was performed using the R package GenABEL (Aulchenko et al., 2007). Genome-wide rapid association studies, using the mixed model and regression (GRAMMAR) method, initially estimate the residuals from the LMM on the assumption that the SNPs have no effect (null model). Subsequently, GRAMMAR then treats the residuals as phenotypes for further genome-wide analysis, using a standard linear mixed model (Zhou and Stephens, 2012). A total of 685 lines were genotyped using the presence/absence Diversity Arrays Technology genotyping-by-sequencing (SillicoDArTs™) platform. By applying zero mismatches and gaps, as well as a stringent alignment using BLASTN (Altschul et al., 1990), we were able to uniquely anchor 15,146 SNPs to a single position of the wheat Chinese Spring reference genome (RefSeq v1.0). The allelic association was calculated for NDVI, after accounting for population structure by implementing the first principal component, as well as the genome-wide kinship matrix for the genotypic trait values of NDVI and PLA. Prior to analysis, markers with more than 10% missing data or minor allele frequency (MAF) less than 5% were excluded from the analysis. A total number of 9,432 high-quality and polymorphic markers remained for the analysis. The cut-off value for markers being identified as significantly associated with the trait was set at the arbitrary threshold of –log_10_ (*P*) > 3 which corresponds to a *p* cut-off of 0.001 significance level. The phenotypic variation explained by a given QTL (PVE) was calculated separately, according to Shim et al. (2015).

This study estimated broad sense (*H*^2^) and narrow sense (*h*^2^) heritability by using the R package sommer (Covarrubias-Pazaran, 2016). A marker-based approach to estimate *σ^2^_*A*_, σ^2^_*D*_*, by calculating additive and dominance relationship matrices, was applied. The models to estimate *h*^2^ and *H*^2^ are given in Eqs. 2, 3, respectively.


(2) $$h^{2}=\frac{\sigma_{A}^{2}}{\sigma_{P}^{2}}$$

(3) $$H^{2}=\frac{\sigma_{A}^{2}+\sigma_{D}^{2}}{\sigma_{P}^{2}}$$

With *σ^2^_*A*_* as the additive genetic variance, *σ^2^_*D*_* as the dominance genetic variance, and *σ^2^_*P*_* as the phenotypic variance.

## Results

### Phenotypic Characterisation of Early Vigour

#### Expression of Early Vigour in the Field

**FIGURE 2:**
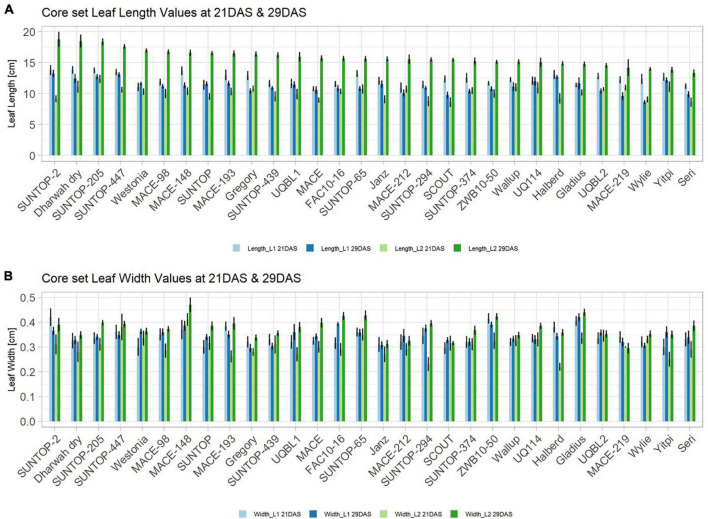
Core set leaf parameters recorded under field conditions. **(A)** Showing leaf length values captured at 21 DAS and 29 DAS and **(B)** showing leaf width values captured at 21 DAS and 29 DAS. Error bars represent standard error.

**FIGURE 3:**
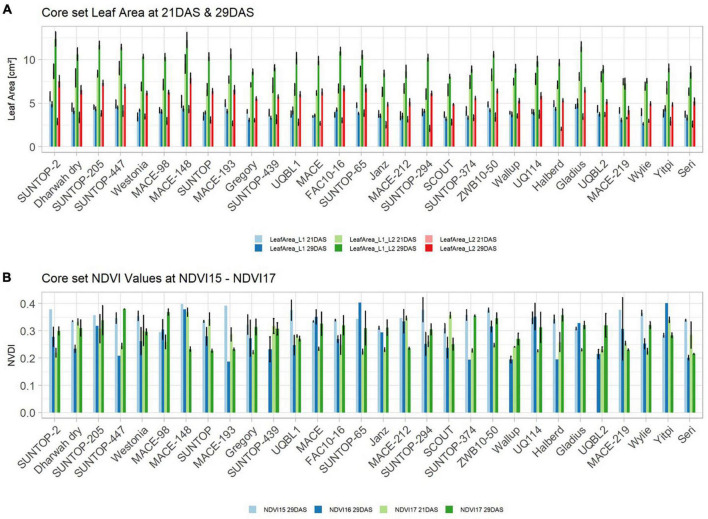
**(A)** Core set leaf area parameters showing leaf area of leaf 1 (L1), leaf 2 (L2) as well as total leaf area (TLA) measured at 21 DAS and 29 DAS, respectively. **(B)** Core set NDVI parameters from NDVI15, NDVI16, and NDVI17 at 21DAS as well as NDVI17 at 29DAS. Error bars represent standard error.

**FIGURE 4:**
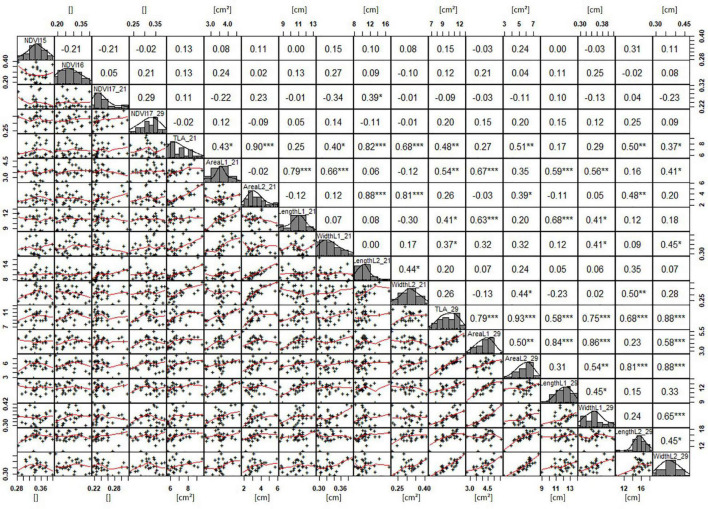
NDVI and leaf parameters captured for the core set of 30 lines in field trials from 2015 to 2017. For each trait, the population distribution is displayed in the centre diagonal. The upper right shows the Pearson correlation coefficient for each trait combination. The lower left half shows the scatter plot with fitted line for each trait combination. * significant at *p* < 0.05; ** significant at *p* < 0.005; *** significant at *p* < 0.001.

Basic descriptive statistical indicators (minimum, maximum, and mean), variation (Var), standard deviation (SD), coefficient of variation (CoV) for NDVI, and leaf parameters of Set 1, Set 2, and the core set are all given in Table 2. For Set 1, the largest mean NDVI values were observed in 2015. For Set 2 and the core set, the largest mean values were reached in 2016, while in Set 2, the overall maximum mean NDVI was recorded in 2015. All leaf parameters which were measured in the core set showed larger values at 29 DAS compared to those recorded at 21 DAS (Table 2). Within the core set the largest values for L1 were reached by Dharwah dry at 21 DAS and SUNTOP-2 at 29 DAS (Figure 2A). Furthermore, a significant (*p* < 0.05) difference between the genotypes was observed at 21 DAS and 29 DAS, with a smaller phenotypical variation at 21 DAS compared to the measurement at 29 DAS (Supplementary Table 1). For leaf length L2, similar results were observed with significant (*p* < 0.05) phenotypical variation at both time points, with a smaller variation at 21 DAS compared to 29 DAS (Supplementary Table 1). The largest leaf length L2 values were reached by SUNTOP-205 at 21 DAS and SUNTOP-2 at 29 DAS, respectively (Figure 2B). Regarding the leaf length of L1, the largest values were reached by SUNTOP-2 at 21 DAS and by Gladius at 29 DAS. For width L1, only at 29 DAS, a significant (*p* < 0.05) phenotypical variation could be observed (Supplementary Table 1). By comparing leaf area values calculated within the core set, it was observed that SUNTOP-2 showed the largest values for L1 at 21 DAS and 29 DAS, as well as for TLA at 21 DAS. For leaf area, L2 at 21 DAS, 29 DAS and TLA at 29 DAS, MACE-148 showed the largest values (Figure 3). For all leaf area traits which were recorded at 21 DAS, the phenotypical variation is smaller compared to the phenotypical variation at 29 DAS (Supplementary Table 1). In order to examine the impact of each leaf characteristic on the TLA, Pearson correlation coefficients for each factor were estimated (Figure 4). Person correlation analysis revealed a larger significant (*p* < 0.001) correlation between TLA at 21 DAS and leaf area L2 at 21 DAS (*r* = 0.89), than between TLA and leaf area L1 at 21 DAS (*p* < 0.05 *r* = 0.42). A similar correlation can be observed for L1 and L2 at 21 DAS, and L2 parameters at 21 DAS. Notably, a significant (*p* < 0.05) correlation was observed between NDVI 17 21 DAS and leaf length L2 at 21 DAS (*r* = 0.39). Furthermore, the relationship between the leaf area of L1 and L2 at 29 DAS and leaf parameters at 29 DAS shows similarities to the calculation made at 21 DAS. Leaf area L1 at 29 DAS shows stronger correlations with leaf TLA at 29 DAS (*r* = 0.79), compared to 21 DAS. However, the L2 area at 29 DAS (*r* = 0.93) shows a stronger correlation to TLA at 29 DAS. Interestingly, the correlation between area L1 and L2 at 21 DAS, and the L1 and L2 at 29 DAS (*r* = 0.47) is slightly smaller than the correlation with L2 area at 29 DAS (*r* = 0.51) and leaf length L2 at 29 DAS.

**TABLE 2:** Descriptive statistics for collected field data for Set 1 (685 lines) and the subsets of lines Set 2 (210 lines) and core set (30 lines) captured at 21 and 29 days after sowing (DAS).

Set	Parameter	Unit	DAS	Year	Descriptive statistics				
					Mean	Min	Max	SD	CoV
Set 1	NDVI		29	2015	0.343	0.26	0.449	0.17	0.5
11	NDVI		29	2016	0.259	0.14	0.447	0.2	0.77
Set 2	NDVI		29	2015	0.341	0.26	0.456	0.03	0.09
12	NDVI		29	2016	0.339	0.27	0.461	0.04	0.11
13	NDVI		21	2017	0.325	0.22	0.394	0.04	0.12
14	NDVI		29	2017	0.264	0.19	0.416	0.03	0.13
Core Set	NDVI		29	2015	0.344	0.28	0.398	0.03	0.08
15	NDVI		29	2016	0.276	0.19	0.403	0.06	0.22
16	NDVI		21	2017	0.245	0.22	0.317	0.03	0.11
17	NDVI		29	2017	0.321	0.24	0.391	0.04	0.13
18	TLA	[cm^2^]	21	2017	6.973	5.7	9.609	1.08	0.16
19	Leaf area L1	[cm^2^]	21	2017	3.569	2.67	4.658	0.48	0.13
20	Leaf area L2	[cm^2^]	21	2017	3.404	1.82	5.904	0.98	0.29
21	Length L1	[cm]	21	2017	10.83	8.59	13.12	1.11	0.1
22	Width L1	[cm]	21	2017	0.33	0.3	0.39	0.03	0.08
23	Length L2	[cm]	21	2017	10.68	8.15	16.96	1.88	0.18
24	Width L2	[cm]	21	2017	0.315	0.22	0.41	0.05	0.15
25	TLA	[cm^2^]	29	2017	9.971	6.66	12.78	1.46	0.15
26	Leaf area L1	[cm^2^]	29	2017	4.221	2.75	5.62	0.63	0.15
27	Leaf area L2	[cm^2^]	29	2017	5.75	3	7.793	1.04	0.18
28	Length L1	[cm]	29	2017	12.04	9.01	13.85	1.04	0.09
29	Width L1	[cm]	29	2017	0.349	0.3	0.42	0.03	0.09
30	Length L2	[cm]	29	2017	15.49	10.3	18.33	1.6	0.1
	Width L2	[cm]	29	2017	0.369	0.29	0.47	0.04	0.12

*Shown are the set of lines examined (Set), parameter examined, unit (NDVI is an index without units), time of measurement in days after sowing (DAS), year of experiment as well as for each set examined, mean value, minimum (Min), maximum (Max), variance (Var), standard deviation (SD), and coefficient of variation (CoV).*

#### Expression of Early Vigour Under Controlled Conditions

**FIGURE 5:**
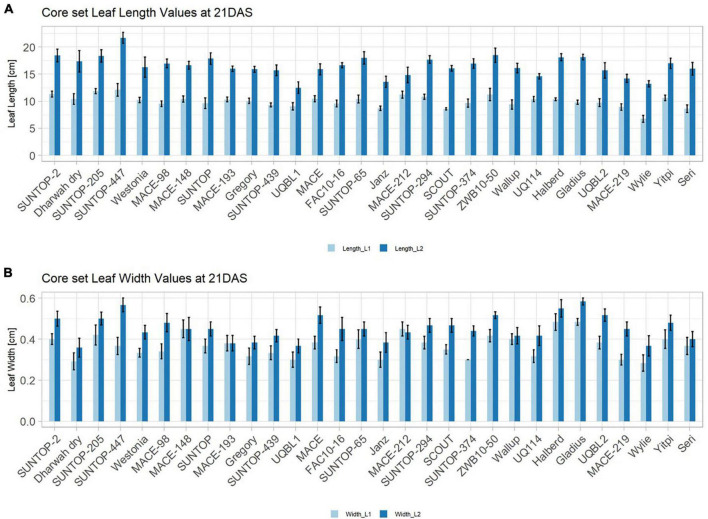
Core set leaf parameters recorded under controlled conditions. **(A)** Showing leaf length values captured at 21 DAS and **(B)** showing leaf width values captured at 21 DAS. Error bars represent standard error.

**FIGURE 6:**
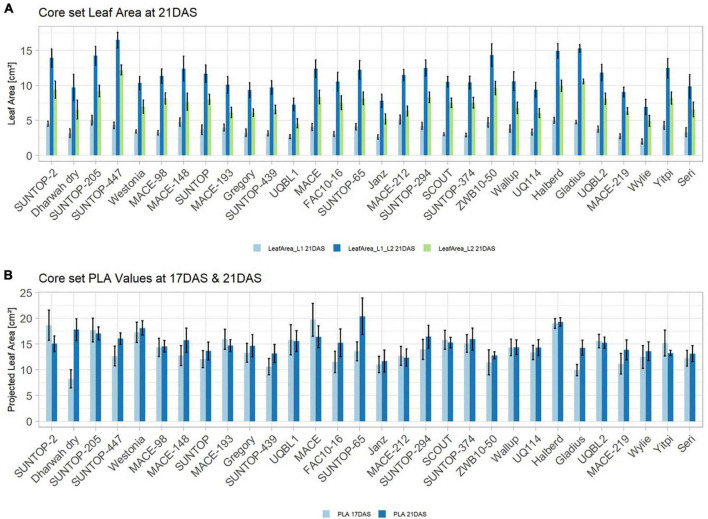
**(A)** Core set leaf area parameters showing leaf area of leaf 1 (L1), leaf 2 (L2) as well as total leaf area (TLA) measured at 21 DAS in greenhouse experiment in 2017. **(B)** Core set projected leaf parameters (PLA) recorded at 17DAS and 21DAS. Error bars represent standard error.

**FIGURE 7:**
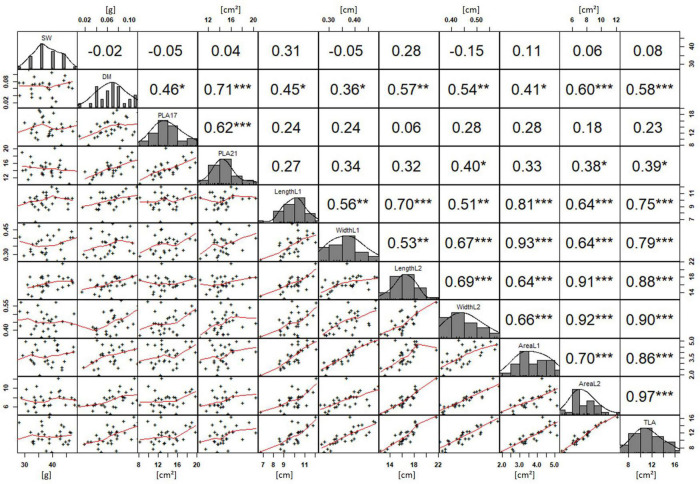
Projected leaf area (PLA) by green pixel count method and leaf parameters captured for core set in greenhouse experiment. For each trait, the population distribution is displayed in the centre diagonal. The upper right shows the Pearson correlation coefficient for each trait combination. The lower left half shows the scatter plot with fitted line for each trait combination. * significant at *p* < 0.05; ** significant at *p* < 0.005; *** significant at *p* < 0.001.

In order to understand how EV can be determined under controlled conditions, this study conducted a trial testing Set 2 as well as the core set within a GH environment. Basic statistical indicators are given in Table 3. Figure 5A reveals that the leaf length and leaf width of L2 exceeds L1 for almost all genotypes at 21 DAS. The only exceptions are the leaf widths of MACE-148 and of MACE-193, where L1 and L2 had similar values (Figure 5B). The largest leaf length values for L1 and L2 were reached by SUNTOP-447 (Figure 5A). As in field conditions, Gladius reached the largest values for leaf width L1 as well as for leaf width L2 (Figure 5B). Furthermore, for all the measured leaf parameters, significant (*p* < 0.05) differences can be observed. However, the recorded phenotypical differences show a lower variation compared to the field conditions (Supplementary Table 2). The calculated leaf area showed a significant (*p* < 0.05) phenotypical difference, particularly the L2, as well as the L1&L2 values (Supplementary Table 2). The SUNTOP-447 exhibited the largest values for leaf length, leaf area L2, and L1&L2, respectively. Maximum leaf area and L1 were exhibited by MACE-212 (Figure 6A). Only around two-thirds of the core set showed increased PLA at 21, compared to 17 DAS (Figure 6B). Pearson coefficients of correlation showed a significant positive relationship of the collected dry matter with the PLA 17 DAS (*p* < 0.05; *r* = 0.46) and with PLA 21 DAS (*p* < 0.001; *r* = 0.71). Furthermore, the PLA at 21 DAS shows a positive correlation to leaf width L2 (*r* = 0.34), as well as to area L2 (*r* = 0.38) and TLA (*r* = 0.39). As under field conditions, the TLA of L1 and L2 is more affected by area L2 (*r* = 0.96). However, area L1 (*r* = 0.85) seems to have a greater impact on TLA in the GH compared to the field. Furthermore, it is noteworthy that dry mass measured at harvest at 21 DAS shows a positive correlation to all captured leaf characteristics. Moreover, seed weight (SW) showed no significant correlation to any other trait (Figure 7).

**TABLE 3:** Descriptive statistics for collected GH data for core set (30 lines) captured at 17 and 21 days after sowing (DAS).

Set	Parameter		DAS	Year	Descriptive statistics					
					Mean	Min	Max	Var	SD	CoV
Core Set	Seed weight	[g]	–	2017	0.095	0.068	0.121	0.000	0.012	0.129
31	PLA	[cm^2^]	17 DAS	2017	13.895	8.236	19.681	7.740	2.913	0.210
32	PLA	[cm^2^]	21 DAS	2017	14.795	10.635	20.348	4.350	2.158	0.146
33	Biomass	[g]	21 DAS	2017	0.067	0.010	0.108	0.001	0.024	0.364
34	TLA	[cm^2^]	21 DAS	2017	11.254	6.915	16.493	5.659	2.518	0.224
35	Leaf area L1	[cm^2^]	21 DAS	2017	3.707	1.983	5.125	0.714	0.904	0.244
36	Leaf area L2	[cm^2^]	21 DAS	2017	7.548	4.600	12.203	2.933	1.799	0.238
37	Length L1	[cm]	21 DAS	2017	9.943	6.750	12.100	1.239	1.273	0.128
38	Width L1	[cm]	21 DAS	2017	0.366	0.267	0.483	0.003	0.060	0.164
39	Length L2	[cm]	21 DAS	2017	16.445	12.500	21.700	3.487	2.034	0.124
	Width L2	[cm]	21 DAS	2017	0.452	0.358	0.583	0.004	0.063	0.138

*Shown are the set of lines examined (Set), parameter examined, unit, time of measurement (DAS), year of experiment as well as mean value, minimum (Min), maximum (Max), variance (Var), standard deviation (SD), and coefficient of variation (CoV).*

#### Components of Early Vigour

**FIGURE 8:**
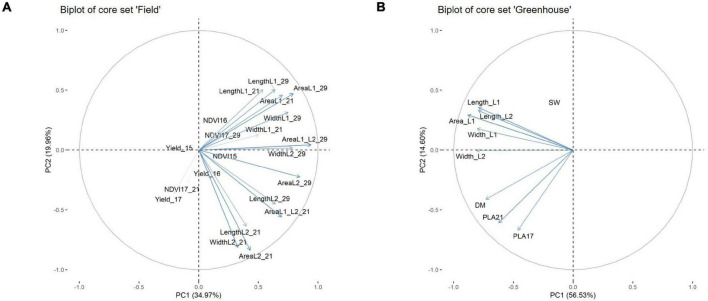
Biplot from principal component analysis for core set field **(A)**, core set greenhouse **(B)**.

To determine which physiological parameters of the leaf had the greatest influence on EV, a principal component (PC) analysis was performed. The analysis was conducted for the core set data and included parameters such as the leaf measurements at 21 DAS and at 29 DAS in the field, or at 21 DAS in the GH, as well as dry matter content, grain weight, and the respective NDVI values from 2015 to 2017 (Figure 8). For the field data, the NDVI in 2015 (NDVI 15) was largely associated with parameters connected to L2 at 21 DAS, and to leaf length and area of L2 at 29 DAS. The NDVI 16 and NDVI 17 at 29 DAS showed a strong association with area L1 at 21 DAS and area L1 at 29 DAS. In this regard, it became apparent that several parameters recorded at 29 DAS, such as area L1 and L2 and leaf width L2, were strongly correlated as well. Interestingly, for NDVI at 21 DAS, no association to any physiological parameters of the leaf was observed. In the GH, it could be observed that area L1 and L2 were slightly and closely more associated with area L2 than with area L1. Furthermore, area L2 appeared to be in a stronger association to leaf width L2 than to leaf length L2. The PLA at 17 DAS and PLA at 29 DAS showed a strong association to each other. However, apart from dry mass, no significant associations to any of the leaf parameters were observed.

### Identifying Genetic Determinants of Early Vigour

**FIGURE 9:**
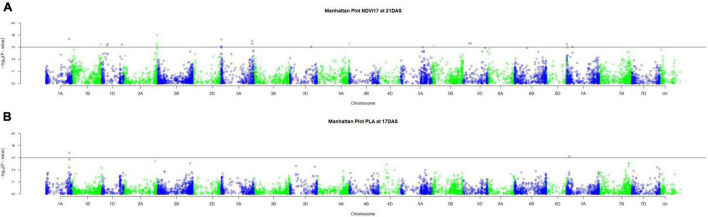
Manhattan plots for **(A)** NDVI17 at 21DAS and **(B)** PLA at 17DAS. The horizontal line at –log_10_ (*P*) = 3 indicates the chosen probability threshold to indicate a significant association with the trait.

Genetic analysis was performed with NDVI values for the 685 genotypes from Set 1 in 2015 (NDVI’15) and in 2016 (NDVI’16), along with NDVI values from 2015 to 2017, and the PLA data from the GH trial in 2017 in the 221 genotypes of Set 2. Several SNP markers exceeded the arbitrary threshold of association (–log_10_ (*P*) = 3) for NDVI and PLA across all years in the genome-wide association study. A total of 41 QTL were associated [–log_10_ (*P*) = 3] with either NDVI or PLA. The majority of trait-associated SNP markers (21) were associated with NDVI at 21 DAS in 2017 in Set 2 (Figure 9). Chromosome positions of all identified marker-trait associations for NDVI and PLA in the different environments are summarized in Table 4. All the identified QTL PVE was low and was ranged between 0.024 to 1.35%. The five most significant QTL, *QSG.qwr-3B.1*, *QSG.qwr-2A.3*, *QSG.qwr-3D.1*, *QSG.qwr-1A.1*, and *QSG.qwr-5B.2* accounted for 0.4% of the variation (Table 4). Most QTL effects were small and few, as QTL were detected in multiple environments or across traits, reflecting the genetic complexity and the strong environmental dependency of EV traits. This finding is consistent with several other studies that have also identified multiple QTL for several EV related traits located on different chromosomes (Table 5). In accordance with this observation, the narrow-sense heritability of both NDVI in Set 1 was found to be moderate with *h*^2^ = 0.22–0.28, and small for NDVI and PLA in Set 2 with *h*^2^ = 0.08–0.04 (Table 6).

**TABLE 4:** QTL identified with significant association to phenotypic traits (*P*-value > −log10 (*P*) = 3).

QTL name	Chr	Position [bp]	Trait	DAS	Year	*P*-value	PVE	No. of reported genes in ± 20kb	Gene-ID	Start position [bp]	End position [bp]
QSG.qwr-1A.1	1A	530870345	NDVI	21	2017	3.68	0.135	1	TraesCS1A02G341200	530883540	530884786
QSG.qwr-1A.2	1A	537895686	PLA	17	2017	3.39	0.125	–	NA	NA	NA
QSG.qwr-2A.1	2A	758570584	NDVI	21	2017	3.17	0.114	3	TraesCS2A02G552800	758553979	758559790
									TraesCS2A02G552900	758583247	758587695
									TraesCS2A02G553000	758588329	758589199
QSG.qwr-2A.2	2A	768989573	NDVI	21	2017	3.34	0.120	3	TraesCS2A02G573200	768981429	768983083
									TraesCS2A02G573300	768996829	768998744
									TraesCS2A02G573400	769002934	769004935
QSG.qwr-2A.3	2A	769344238	NDVI	21	2017	4	0.152	1	TraesCS2A02G574300	769340976	769344226
QSG.qwr-2A.4	2A	775175704	NDVI	21	2017	3.06	0.113	1	TraesCS2A02G583000	775165226	775166200
QSG.qwr-3A.1	3A	8267133	NDVI	21	2017	3.08	0.107	2	TraesCS3A02G008800	8253788	8255463
									TraesCS3A02G008900	8258758	8260510
QSG.qwr-3A.2	3A	9212918	NDVI	21	2017	3.64	0.134	1	TraesCS3A02G011400	9210008	9210976
QSG.qwr-3A.3	3A	9463808	NDVI	21	2017	3.03	0.107	3	TraesCS3A02G011800	9444895	9445740
									TraesCS3A02G011900	9473058	9473828
									TraesCS3A02G012000	9480858	9481594
QSG.qwr-3A.4	3A	711365648	NDVI	21	2017	3.5	0.122	1	TraesCS3A02G480500	711380680	711385803
	3A	711366350	NDVI	21	2017	3.27		–	NA	NA	NA
QSG.qwr-4A.1	4A	744471786	NDVI	21	2017	3.28	0.119	1	TraesCS4A02G499600	744456472	744458084
QSG.qwr-5A.1	5A	526386152	NDVI	29	2015	3.14	0.115	5	ENSRNA050011196	526378014	526378087
									ENSRNA050021751	526384607	526384709
									TraesCS5A02G315700	526393365	526396850
									TraesCS5A02G315800	526396904	526400499
									TraesCS5A02G315900	526400750	526402134
QSG.qwr-5A.2	5A	529810097	NDVI	29	2015	3.54	0.025	–	NA	NA	NA
QSG.qwr-5A.3	5A	592515889	NDVI	29	2015	3.07	0.030	1	TraesCS5A02G398700	592518737	592520426
QSG.qwr-6A.1	6A	5480626	NDVI	29	2015	3.14		1	TraesCS6A02G011800	5473439	5480862
QSG.qwr-7A.1	7A	263934	NDVI	21	2017	3.24	0.112	2	TraesCS7A02G000200	242359	246258
									TraesCS7A02G000300	250565	253987
QSG.qwr-7A.2	7A	51498553	PLA	17	2017	3.1	0.112	–	NA	NA	NA
QSG.qwr-7A.3	7A	130596094	NDVI	21	2017	3.03	0.106	1	TraesCS7A02G177400	130608975	130613414
QSG.qwr-7A.4	7A	694049656	NDVI	29	2016	3.63	0.040	2	TraesCS7A02G506800	694053373	694053943
									TraesCS7A02G506900	694060394	694067693
QSG.qwr-1B.1	1B	37762680	NDVI	29	2016	3.12	0.037	–	NA	NA	NA
QSG.qwr-1B.2	1B	84949441	NDVI	29	2016	3.47	0.039	–	NA	NA	NA
QSG.qwr-1B.3	1B	658902240	NDVI	21	2017	3.24	0.113	3	TraesCS1B02G434300	658908133	658910735
									TraesCS1B02G434400	658911233	658914898
									TraesCS1B02G434500	658915051	658919932
QSG.qwr-3B.1	3B	22137315	NDVI	29	2016	4.24	0.051	1	TraesCS3B02G042800	22129256	22130588
QSG.qwr-5B.1	5B	52306024	NDVI	21	2017	3.06	0.114	1	TraesCS5B02G046800	52317367	52318139
QSG.qwr-5B.2	5B	162778081	NDVI	29	2015	3.64	0.030	1	TraesCS5B02G110800	162771499	162776936
QSG.qwr-5B.3	5B	576298790	NDVI	29	2015	3	0.024	–	NA	NA	NA
QSG.qwr-7B.1	7B	748033995	NDVI	29	2015	3.27	0.026	1	TraesCS7B02G497400	748012557	748015135
QSG.qwr-1D.1	1D	52680610	NDVI	29	2016	3.2	0.035	1	TraesCS1D02G072300	52658816	52664518
QSG.qwr-1D.2	1D	111980607	NDVI	21	2017	3.15	0.112	1	TraesCS1D02G116200	111967743	111980590
QSG.qwr-1D.3	1D	134460288	NDVI	21	2017	3.28	0.120	–	NA	NA	NA
QSG.qwr-1D.4	1D	461051177	NDVI	21	2017	3.21	0.112	1	TraesCS1D02G389300	461052237	461057083
QSG.qwr-2D.1	2D	19551003	NDVI	29	2016	3.19	0.035	3	TraesCS2D02G051300	19543541	19544843
									TraesCS2D02G051400	19555735	19557093
									TraesCS2D02G051500	19561151	19563368
QSG.qwr-2D.2	2D	69503900	NDVI	29	2017	3.05	0.100	2	TraesCS2D02G120100	69502005	69504329
									TraesCS2D02G120200	69504557	69510365
QSG.qwr-2D.3	2D	362486328	NDVI	29	2017	3.11	0.108	–	NA	NA	NA
QSG.qwr-3D.1	3D	325183855	NDVI	29	2015	3.76	0.032	–	NA	NA	NA
QSG.qwr-3D.2	3D	481926113	NDVI	21	2017	3.04	0.113	1	TraesCS3D02G368700	481908948	481926630
QSG.qwr-5D.1	5D	138341788	NDVI	21	2017	3.31	0.115	–	NA	NA	NA
QSG.qwr-5D.2	5D	179786153	NDVI	21	2017	3.3	0.115	1	TraesCS5D02G123000	179762666	179778583
QSG.qwr-6D.1	6D	50627689	NDVI	29	2015	3.35	0.027	–	NA	NA	NA
QSG.qwr-7D.1	7D	533491186	NDVI	29	2015	3.11	0.026	–	NA	NA	NA

*Shown are QTL identifier, chromosome location (Chr), Positions refer to physical positions [bp] on reference genome Chinese Spring (RefSeq v1.0), trait, time of measurement (DAS), year of measurement (Year), probability of association with trait by chance [−log10 (p-value)], phenotypic variation explained by a given QTL (PVE), number of genes with 20 kb of the QTL SNP location (No. of genes ± 20 kb), name of genes reported within the 20 kb radius of the QTL SNP (Gene-ID), start and end position of reported gene in [bp] (Start Position [bp]/End Position [bp]).*

**TABLE 5:** Summary of early vigour related QTL reported in previous publications.

Trait	QTL/marker name	Chr	Publication
Coleoptile length	QClp.ipk-1A	1A	Landjeva et al., 2008
Coleoptile length	QClp.ipk-1B	1B	
Coleoptile length	ksuG9c	1A	Rebetzke et al., 2007
Coleoptile length	Stm55ltgag	2D	
Coleoptile length	psr426	5A	
Coleoptile length	psr326b	5D	
Embryo size	gwm18	1B	Moore and Rebetzke, 2015
EV, canopy temperature	41	3B	Bennet et al., 2012
Ground cover	QGCw.caas-1A.1	1A	Li et al., 2014
Ground cover	QGCw-caas-1D	1D	
Ground cover	QGCs-caas-2A.2	2A	
Ground cover	QGCs.caas-3B.1	3B	
Ground cover	QGCw-caas-5B	5B	
Ground cover	QGCw.caas-5B	5B	
Ground cover	QGCwcaas-5D	5D	
Ground cover	QGCscaas-6A	6A	
Ground cover	QGCs-caas-6A	6A	
Leaf length	gwm261	2D	Moore and Rebetzke, 2015
Leaf length	cdo669b	4B	
Leaf length	E36/M60-210-P1	2D	Steege et al., 2005
Leaf length	E48/M48-217-P2	5D	
Leaf length	E48/M60-225-P1	6D	
Leaf length	E45/M52-150-P1	7D	
Leaf width	wmc190	2D	Moore and Rebetzke, 2015
Leaf width	wmc289	5B	
Leaf width	E45/M52-274-P1	1D	Steege et al., 2005
Leaf width	Xgwm458	1D	
Leaf width	E42/M51-482-P2	2D	
Leaf width	Xgwm165	4D	
Leaf width	E42/M52-241-P1	5D	
Leaf width	E51/M52-189-P1	7D	
NDVI	QNDVIs-caas-3A	3A	Li et al., 2014
NDVI	QNDVIw-caas-6D	6D	
NDVI	QYld.aww-1B.2	1B	Tura et al., 2020
NDVI	QTgw.aww-1B	1B	
Relative growth rate	QRgr.saas-5A	5A	Li et al., 2017
Root dry weight	QRdw.saas-5A	5A	
Root length	QRlp.ipk-1A	1A	Landjeva et al., 2008
Root length	QRlp.ipk-7D	7D	
Shoot biomass	Rht-B1	4B	Ryan et al., 2015
Shoot biomass	Rht-D1	4D	
Shoot biomass	wmc525	7A	
Shoot fresh weight	QSfw.saas-5A	5A	Li et al., 2017
Shoot dry weight	QSdw.saas-5A	5A	
Total leaf area	QTla.saas-5A	5A	

*Reported trait (Trait), name of the respective QTL or marker that is associated with the trait (QTL/Marker Name), chromosome on which the QTL or marker is located (Chr), cited publication which reported QTL (Publication).*

**TABLE 6:** Narrow-sense (h2) and broad-sense heritability for NDVI and PLA recorded in set 1 (685 lines) and set 2 (210 lines).

Set	Trait	DAS	Year	Heritability	
				h^2^	H^2^
Set 1	NDVI	29	2015	0.22	0.28
	NDVI	29	2016	0.28	0.38
Set 2	NDVI	29	2015	0.05	0.06
	NDVI	29	2016	0.04	0.05
	NDVI	21	2017	0.07	0.08
	NDVI	29	2017	0.06	0.07
	PLA	17	2017	0.04	0.04

*Trait, time of measurement (DAS), year of measurement (Year), and narrow sense heritability (h^2^) as well as broad sense heritability (H^2^).*

## Discussion

The first objective of this study was to provide information on physiological components that contribute to EV of wheat, both in the field and under controlled conditions. Several studies observed a strong contribution of leaf width to a specific leaf area, and consequently to EV (Rebetzke and Richards, 1999; Richards and Lukacs, 2002; Maydup et al., 2012). Our results showed that the variation in EV is strongly associated with the leaf length of both L1 and L2 in the field, as well as in the GH. For both leaves (L1 and L2), leaf length is the main contributor to a larger leaf area development, and, consequently, to an increased EV. Furthermore, leaf length of L2 showed a slightly greater impact on the area of L1 and L2 with an advancing development. This finding agrees with other studies reporting a significant positive correlation between the area of L2 and the leaf length (Nursinow et al., 2011; Boden et al., 2014; Moore and Rebetzke, 2015; Duan et al., 2016). The data revealed faster development of L2 in the GH compared to the field studies. This was also observed by Rebetzke et al. (2007) and leads to the conclusion that EV can be recorded at earlier stages under controlled conditions than in the field. Previous studies have reported that embryo size is a highly heritable trait that is strongly associated with leaf area (López-Castañeda et al., 1996; Rebetzke and Richards, 1999; Aparicio et al., 2002; Moore and Rebetzke, 2015). Since it has been established that embryo size increases with seed weight in wheat (Moore and Rebetzke, 2015) and barley (López-Castañeda et al., 1996), this study used seed weight to indirectly evaluate the impact of embryo size on EV. In the GH experiment, it was not possible to demonstrate the positive impact of embryo size on EV by using seed weight, since no significant correlation with any measured leaf parameter was observed. Nonetheless, several studies reported a positive effect of the embryo size on early vigour in wheat (Rebetzke and Richards, 1999; Richards and Lukacs, 2002; Moore and Rebetzke, 2015). In other studies, however, the total variation in EV could not be exclusively explained by the considering seed weight (Maydup et al., 2012), and the seed density has also been suggested as a potentially more decisive factor in determining EV (Ball et al., 2011). This partially explains why no correlation between seed weight and EV parameters could be found in the present study. In the GH experiment, biomass was recorded and was exhibited as a significant correlation with the area of L2, as well as area of L1 and L2, suggesting an increased above-ground biomass for genotypes with greater EV. Richard et al. (2019) reported that lines with increased above-ground biomass were strongly associated with an increased grain yield.

The second objective of this study was to evaluate high-throughput methods to precisely record physiological leaf characteristics in field and greenhouse trials. Mullan and Reynolds (2010) reported a significant correlation between NDVI, leaf area, and biomass. These PCA results from the current study tended to confirm that NDVI and the leaf area are related. Although the biomass in the field was not separately measured, a significant positive correlation between biomass and leaf area in the GH experiment suggested a putative relationship. In the correlation analysis, only one significant correlation between NDVI and leaf parameters could be established, which was NDVI17 at 21 DAS and leaf length L2 at 21 DAS. This confirms that leaf length L2 contributes more to an increased EV compared to leaf dimensions of L1. This is also supported by other studies (López-Castañeda et al., 1996; Richards and Lukacs, 2002; Duan et al., 2016). In terms of high-throughput phenotyping methods for EV in GH environments, this study tested the ability of a green pixel counter as a low-cost method. Since embryo size strongly affects EV, the method is not able to explain the trait completely. However, the results for PLA calculated by the green pixel counter indicate great potential to measure EV under controlled conditions. In particular, the leaf parameters of L2 showed a significant correlation with PLA, as well as with the biomass. Furthermore, the green pixel counter was successfully used to measure coleoptile tiller length, a trait which strongly affects EV and is also highly correlated to embryo size.

The third aim of this study was to achieve a better understanding of EV genetics in wheat. The GWAS for Set 1 and Set 2 revealed 41 SNP markers for NDVI and for PLA, which were linked to 60 protein-coding regions across 17 chromosomes. Consistent with previous studies, the present study shows that EV in wheat is a quantitative trait with numerous QTL located across several chromosomes (Li et al., 2014; Moore and Rebetzke, 2015; Boudiar et al., 2016). Numerous studies have reported the effect of dwarfing genes on coleoptile length (Rebetzke et al., 2007; Li et al., 2017), coleoptile width (Rebetzke et al., 2014), and leaf epidermal cell dimension (Botwright et al., 2005). In most of these studies, the influence of dwarfing genes was highlighted. The gibberellic acid (GA)-insensitive dwarfing genes *Rht B1b on* chromosome 4B and *Rht D1db* on chromosome 4D have been reported to reduce coleoptile length and, consequently, EV, since they decrease epidermal cell length in leaf tissue (Ellis et al., 2004; Rebetzke et al., 2007; Yu and Bai, 2010; Li et al., 2017; Rebetzke et al., 2017). In our study, no SNP markers were detected on either one of the 4B or 4D chromosomes. This suggests that NDVI and PLA are the less affected traits by the presence of *Rht B1b* and of *Rht D1b*. Comparable results were reported in Li et al. (2014), were none of the parental lines that carried *Rht-B1b*, and only one parental line contained *Rht-D1b*, while no QTL was identified on chromosome 4D. However, a significant correlation could be observed between *Rht-D1* and NDVI and the ground cover in certain environments. The *Rht 8*, on the short arm of chromosome 2D, is a GA-responsive dwarfing gene reported to have a secondary effect of reducing epidermal cell length. Hence, it is more appropriate for achieving good canopy cover in combination with a semi-dwarf growth habit (Botwright et al., 2005). Chai et al. (2019) reported the WRKY transcription factor *TraesCS2D01G051500* as a possible candidate gene for *Rht8*. We identified QTL *QSG.qwr-2D.1* in the vicinity of *TraesCS2D01G051500*, and found this QTL to be significantly associated with NDVI17 at 29 DAS. Several studies have identified QTL associated with coleoptile length on chromosome 1B, including markers *XpGTG-mTCGA294* (Yu and Bai, 2010) and *wsnp_CAP11_c2596_1325540* (Ma et al., 2020). This study confirms that chromosome 1B is a region of interest for EV, since three significant QTL were detected on this chromosome. That applies particularly to *QSG.qwr-1B*, which is located at the same region on the long arm region of chromosome 1B as *wsnp_CAP11_c2596_1325540*, as reported in Ma et al. (2020). Another region of interest is chromosome 5A, which is also considered as the most important chromosome for stay-green traits (Shi et al., 2017; Liu et al., 2019), including the isopentenyl transferase gene (Gan and Amasino, 1995). Furthermore, chromosome 5A harbours several major developmental genes, such as the vernalisation gene *Vrn1*, frost resistance gene *Fr1*, as well as genes for ear emergence time and for the plant height (Sutka and Snape, 1989; Kato et al., 1999; Galiba et al., 1995).

The results of the current phenotypic investigation extended our insights into the EV trait in wheat. The key characteristics of EV and the relationship with other traits, such as biomass, were successfully identified. In addition, this study presents effective methods that can be used to detect EV in the field, as well as under controlled conditions. In particular, the connection between the leaf length parameters and the NDVI highlights the great potential of NDVI, especially if given recent advances in unmanned aerial vehicle or drone phenotyping platforms (Shi et al., 2016). Nevertheless, potential interactions due to environmental factors must be clarified by practical crop management for a better understanding, since factors such as sowing time, sowing depth, sowing rate, and row spacing may also influence EV. In particular, the interaction and value of EV in specific target environments must be clarified. Furthermore, it has to be considered that yield predictions based on EV can be very challenging. Since the trait is recorded at a very early developmental stage, its relationship to yield performance can subsequently be influenced by a multitude of complex environmental factors. For example, abiotic stress factors have a particularly decisive influence on yield and its yield components, especially during key developmental stages, such as tillering and flowering. Nevertheless, EV is essential for good crop establishment and, therefore, can impact yield even at this early stage. Hence, we suggest incorporating EV measurements into experiments by using the NDVI data in performance evaluations, such as stay-green trials. Our genomic analysis has identified QTL that is associated with EV, which are co-located or are closely linked to key genes controlling the plant development, such as plant height, coleoptile length, stay-green, and vernalisation. The results support the theory that EV is a trait regulated by pleiotropic genes. These findings may help identify the key drivers and determine potential trade-offs with important agronomic traits. Given that the trait is underpinned by many QTL with small effects, marker-assisted selection or gene-based approaches are likely to be challenging; however, genomic prediction approaches provide a suitable option for future breeding.

## Data Availability Statement

The original contributions presented in the study are included in the article/Supplementary Material, further inquiries can be directed to the corresponding author.

## Author Contributions

JC and LTH conceived the study. SV, SA, and JC performed the experiments. SV and AS analysed the phenotypic data. SV wrote the manuscript with further input from AS, RJS, JC, SA, and LTH. All authors contributed to the article and approved the submitted version.

## Conflict of Interest

The authors declare that the research was conducted in the absence of any commercial or financial relationships that could be construed as a potential conflict of interest.

## Publisher’s Note

All claims expressed in this article are solely those of the authors and do not necessarily represent those of their affiliated organizations, or those of the publisher, the editors and the reviewers. Any product that may be evaluated in this article, or claim that may be made by its manufacturer, is not guaranteed or endorsed by the publisher.

## Footnotes

1
http://CRAN.R-project.org/package=lme4

2
https://cran.r-project.org/web/packages/lsmeans/index.html

## Abbreviations

Abbreviations
CoV: coefficient of variation
DAS: days after sowing
EV: early vigour
GH: greenhouse
GRAMMAR: genome-wide rapid association using mixed model and regression
GWAS: genome-wide association study
L1: leaf one
L2: leaf two
LMM: linear mixed model
MAF: minor allele frequency
Ma-NAM: Mace derived nested association mapping population
MR-NAM: multi-parent nested association mapping population
N: nitrogen
NDVI: normalised difference vegetation index
PC: principal component
PLA: projected leaf area (measured using the green pixel counter)
Sc-NAM: scout derived nested association mapping population
SD: standard deviation
Su-NAM: Suntop derived nested association mapping population
TLA: total leaf area (= calculated area of L1 + L2)
Var: variance.
