# Supplementary material for: Dissecting the Genetics of Early Vigour to Design Drought-Adapted Wheat
Source: Front Plant Sci. 2022 Jan 3;12:754439. doi: 10.3389/fpls.2021.754439 (PMC8763316; doi:10.3389/fpls.2021.754439)
Supplement: Supplementary file 1 [file Data_Sheet_1.PDF]

frontiers

Supplementary Material

1 Supplementary Tables

Supplementary Table S1: Mean NDVI and leaf parameters for every core set genotype. Genotypes with same letters indicate no significant difference (*p*<0.05) for the particular trait.

Genotype	Trait	NDVI				Leaf Area						Leaf Length				Leaf Width			
						L1	L1	L2	L2	L1 & L2	L1 & L2	L1	L1	L2	L2	L1	L1	L2	L2
	Year	2015	2016	2017	2017	2017						2017				2017			
	DAS	29	29	21	29	21	29	21	29	21	29	21	29	21	29	21	29	21	29
Dharwah dry		0.34 a	0.23 a	0.33 ab	0.31 ab	4.53 ab	4.11 abcd	3.35 ab	6.51 abcde	7.88 a	10.61 abcdef	13.8 a	12.4 abcde	11.07 ab	18.47 ab	0.33 a	0.33 bcd	0.28 abc	0.35 cdef
FAC10-16		0.34 a	0.27 a	0.25 ab	0.36 ab	3.65 ab	4.26 abcd	3.03 ab	6.68 abcde	6.68 a	10.94 abcde	11.52 ab	10.84 bcdefghi	10.33 ab	15.65 bcdefg	0.32 a	0.39 ab	0.29 abc	0.43 abc
Gladius		0.31 a	0.33 a	0.23 ab	0.32 ab	4.61 ab	4.97 a	3.45 ab	6.5 abcde	8.06 a	11.47 abc	11.38 ab	11.65 abcdefgh	10.17 ab	14.73 defg	0.41 a	0.42 a	0.34 abc	0.44 ab
Gregory		0.32 a	0.27 a	0.22 b	0.34 ab	4.05 ab	3.08 de	3.04 ab	5.51 cdef	7.09 a	8.59 defgh	12.86 ab	10.41 cdefghi	10.78 ab	16.33 abcdef	0.32 a	0.3 d	0.28 abc	0.34 def
Halberd		0.34 a	0.19 a	0.26 ab	0.36 ab	4.93 ab	4.34 abcd	2.05 b	5.3 def	7.09 a	9.64 abcdefgh	13.05 ab	12.64 abcd	9.21 ab	14.84 defg	0.38 a	0.34 abcd	0.22 c	0.36 bcdef
UQBL1		0.37 a	0.25 a	NA	NA	3.74 ab	4.13 abcd	2.82 ab	6.02 abcdef	6.56 a	10.15 abcdefg	11.62 ab	11.43 abcdefgh	9.88 ab	15.91 abcdefg	0.32 a	0.36 abcd	0.27 abc	0.38 bcde
Janz		0.31 a	0.29 a	NA	NA	3.77 ab	3.55 bcde	2.51 ab	4.86 ef	6.28 a	8.4 efgh	12.04 ab	11.51 abcdefgh	9.05 b	15.55 cdefg	0.31 a	0.31 cd	0.27 abc	0.31 ef
MACE		0.33 a	0.38 a	0.23 ab	0.31 ab	3.47 b	3.6 abcde	2.68 ab	6.27 abcde	6.15 a	9.87 abcdefgh	10.76 b	10.55 cdefghi	8.9 b	15.68 bcdefg	0.33 a	0.34 abcd	0.3 abc	0.4 abcd

MACE-148	0.4 a	0.19 a	0.37 a	0.23 ab	5.2 ab	4.36 abcd	4.29 a	7.86 a	9.49 a	12.23 a	13.67 ab	11.29 abcdefgh	10.35 ab	16.58 abcdef	0.37 a	0.39 abc	0.41 a	0.47 a
MACE-193	0.39 a	0.33 a	0.29 ab	0.23 ab	5.01 ab	4.07 abcd	2.68 ab	6.53 abcde	7.69 a	10.6 abcdef	12.97 ab	11.62 abcdefgh	10.31 ab	16.44 abcdef	0.38 a	0.35 abcd	0.26 abc	0.4 abcde
MACE-212	0.35 a	0.31 a	0.35 ab	0.24 ab	3.54 b	3.52 bcde	3.14 ab	5.11 def	6.68 a	8.62 cdefgh	10.92 ab	10.07 efghi	10.69 ab	15.52 cdefg	0.32 a	0.35 abcd	0.29 abc	0.33 def
MACE-219	0.38 a	0.3 a	0.25 ab	0.23 ab	4.17 ab	3.07 de	3.29 ab	4.17 f	7.46 a	7.23 h	12.2 ab	9.58 hi	10.96 ab	14.16 efg	0.34 a	0.32 bcd	0.3 abc	0.3 f
MACE-98	0.3 a	0.35 a	0.26 ab	0.37 ab	4.17 ab	4.02 abcd	2.96 ab	6.24 abcde	7.13 a	10.26 abcdefg	11.86 ab	11.13 abcdefgh	9.98 ab	16.72 abcdef	0.35 a	0.36 abcd	0.29 abc	0.37 bcdef
UQ114	0.35 a	0.35 a	0.23 ab	0.31 ab	4.03 ab	3.95 abcde	3.73 ab	5.83 bcdef	7.77 a	9.78 abcdefgh	12.06 ab	11.95 abcdefg	10.89 ab	15.03 defg	0.34 a	0.33 bcd	0.33 abc	0.39 bcde
Scout	0.31 a	0.24 a	0.36 ab	0.25 ab	3.68 ab	3.2 cde	2.83 ab	4.86 ef	6.51 a	8.06 fgh	12.31 ab	9.72 ghi	8.6 b	15.44 defg	0.3 a	0.33 bcd	0.32 abc	0.32 def
Seri	0.34 a	0.2 a	0.28 ab	0.22 ab	3.71 ab	3.34 cde	2.6 ab	5.17 def	6.31 a	8.54 defgh	11.15 ab	9.86 fghi	8.58 b	13.28 g	0.33 a	0.34 abcd	0.29 abc	0.39 bcde
SUNTOP	0.33 a	0.28 a	0.34 ab	0.23 ab	3.49 b	3.95 abcde	3.06 ab	6.37 abcde	6.55 a	10.32 abcdefg	11.39 ab	11.57 abcdefgh	9.5 ab	16.51 abcdef	0.3 a	0.34 abcd	0.32 abc	0.39 bcde
SUNTOP-2	0.38 a	0.32 a	0.22 b	0.31 ab	5.77 a	4.86 ab	2.88 ab	7.49 ab	8.65 a	12.35 a	13.76 a	13.21 a	9.13 b	18.72 a	0.42 a	0.37 abcd	0.31 abc	0.39 abcde
SUNTOP-205	0.36 a	0.25 a	0.31 ab	0.39 a	4.55 ab	4.34 abcd	3.82 ab	7.31 abc	8.37 a	11.65 ab	13.72 ab	12.71 abc	12.35 a	18.33 abc	0.34 a	0.34 abcd	0.31 abc	0.4 abcd
SUNTOP-294	0.38 a	0.19 a	0.26 ab	0.33 ab	3.94 ab	4.1 abcd	2.1 b	6.12 abcde	6.04 a	10.21 abcdefg	11.43 ab	10.91 abcdefghi	8.72 b	15.46 defg	0.35 a	0.38 abcd	0.23 c	0.4 abcde
SUNTOP-374	0.36 a	0.23 a	0.23 ab	0.35 ab	4.11 ab	3.33 cde	3.32 ab	5.57 bcdef	7.43 a	8.89 bcdefgh	12.53 ab	10.36 defghi	10.46 ab	15.22 defg	0.32 a	0.32 bcd	0.31 abc	0.37 bcdef
SUNTOP-439	NA	0.21 a	0.32 ab	0.33 ab	3.9 ab	3.31 cde	3.14 ab	5.75 bcdef	7.05 a	9.06 bcdefgh	11.59 ab	10.9 abcdefghi	9.52 ab	16.18 abcdef	0.33 a	0.3 d	0.31 abc	0.36 cdef
SUNTOP-447	0.35 a	0.4 a	0.24 ab	0.38 a	4.9 ab	4.52 abc	4.11 ab	6.9 abcd	9.01 a	11.42 abcd	13.46 ab	13.03 ab	10.59 ab	17.55 abcd	0.36 a	0.35 abcd	0.38 ab	0.39 abcde

SUNTOP-65	0.34 a	0.28 a	0.22 b	0.31 ab	4.76 ab	3.83 abcde	3.85 ab	6.7 abcde	8.61 a	10.53 abcdef	13.19 ab	10.78 bcdefghi	10.72 ab	15.58 cdefg	0.36 a	0.36 abcd	0.35 abc	0.43 abc
Wallup	NA	0.19 a	0.24 ab	0.27 ab	3.91 ab	3.7 abcde	3.54 ab	5.27 def	7.45 a	8.97 bcdefgh	12.22 ab	11.09 abcdefgh	10.98 ab	15.08 defg	0.32 a	0.33 bcd	0.33 abc	0.35 cdef
Westonia	0.35 a	0.26 a	0.3 ab	0.31 ab	3.43 b	4.2 abcd	3.47 ab	6.15 abcde	6.89 a	10.34 abcdefg	11.06 ab	11.59 abcdefgh	10.31 ab	16.96 abcde	0.3 a	0.36 abcd	0.34 abc	0.36 bcdef
UQBL2	NA	0.21 a	0.23 ab	0.32 ab	4.34 ab	3.74 abcde	3.7 ab	5.13 def	8.04 a	8.87 bcdefgh	12.79 ab	10.42 cdefghi	10.67 ab	14.51 efg	0.34 a	0.36 abcd	0.35 abc	0.35 cdef
Wylie	0.37 a	0.25 a	0.22 ab	0.32 ab	3.96 ab	2.63 e	2.98 ab	4.94 ef	6.94 a	7.56 gh	12.32 ab	8.59 i	9.02 b	13.96 fg	0.32 a	0.31 cd	0.33 abc	0.35 cdef
Yitpi	0.28 a	0.4 a	0.34 ab	0.28 ab	3.83 ab	4.39 abcd	2.92 ab	4.82 ef	6.75 a	9.02 bcdefgh	12.69 ab	12.15 abcdef	11.08 ab	13.78 fg	0.3 a	0.36 abcd	0.25 bc	0.35 cdef
ZWB10-50	0.38 a	0.32 a	0.25 ab	0.35 ab	4.86 ab	4.19 abcd	3.39 ab	6.39 abcde	8.25 a	10.58 abcdef	11.65 ab	10.73 bcdefghi	10 ab	15.11 defg	0.42 a	0.39 ab	0.33 abc	0.42 abc

Supplementary Table S2: Mean PLA and leaf parameters for every core set genotype. Genotypes with same letters indicate no significant difference (*p*<0.05) for the particular trait.

Genotype	Trait	PLA		Leaf Area			Leaf Length		Leaf Width	
				L1	L2	L1 & L2	L1	L2	L1	L2
	DAS	17	21	21	21	21	21	21	21	21
Dharwah-dry		8.24 b	17.77 a	3.15 ab	6.54 bcde	9.69 bcde	10.4 ab	17.37 abc	0.29 b	0.36 b
FAC10-16		11.5 ab	15.21 a	3.03 ab	7.53 abcde	10.55 abcde	9.55 ab	16.63 abc	0.32 ab	0.45 ab
Gladius		9.92 ab	14.25 a	4.72 a	10.57 ab	15.29 ab	9.78 ab	18.15 abc	0.48 a	0.58 a
Gregory		13.27 ab	14.63 a	3.24 ab	6.09 bcde	9.33 bcde	10.05 ab	15.9 bc	0.32 ab	0.38 ab
Halberd		18.97 ab	19.27 a	5:00 AM	9.95 ab	14.95 ab	10.35 ab	18.12 abc	0.48 a	0.55 ab
UQBL1		15.78 ab	15.53 a	2.66 ab	4.6 e	7.26 de	9.05 ab	12.5 c	0.3 ab	0.37 b
Janz		11 ab	11.66 a	2.6 ab	5.19 cde	7.8 cde	8.7 ab	13.57 bc	0.3 ab	0.38 ab
MACE		19.68 a	16.35 a	4.06 ab	8.33 abcde	12.39 abcde	10.43 ab	15.9 bc	0.38 ab	0.52 ab
MACE-148		12.74 ab	15.7 a	4.74 a	7.66 abcde	12.4 abcde	10.38 ab	16.63 abc	0.45 ab	0.45 ab
MACE-193		15.92 ab	14.69 a	3.96 ab	6.14 bcde	10.1 abcde	10.32 ab	16 abc	0.38 ab	0.38 ab
MACE-212		12.67 ab	12.35 a	5.13 a	6.35 bcde	11.47 abcde	11.22 a	14.83 bc	0.45 ab	0.43 ab
MACE-219		11.14 ab	13.84 a	2.72 ab	6.33 bcde	9.05 bcde	8.92 ab	14.22 bc	0.3 ab	0.45 ab
MACE-98		14.33 ab	14.53 a	3.22 ab	8.13 abcde	11.35 abcde	9.52 ab	16.96 abc	0.34 ab	0.48 ab
UQ114		13.36 ab	14.28 a	3.33 ab	6.05 bcde	9.38 bcde	10.4 ab	14.58 bc	0.32 ab	0.42 ab
Scout		15.78 ab	15.25 a	3 ab	7.52 abcde	10.52 abcde	8.58 ab	16.05 abc	0.35 ab	0.47 ab
Seri		12.22 ab	13.11 a	3.3 ab	6.57 bcde	9.87 abcde	8.62 ab	16 abc	0.37 ab	0.4 ab
SUNTOP		12.07 ab	13.61 a	3.67 ab	8 abcde	11.67 abcde	9.62 ab	17.87 abc	0.37 ab	0.45 ab
SUNTOP-2		18.61 ab	15.03 a	4.52 ab	9.39 abcd	13.91 abcd	11.32 a	18.43 ab	0.4 ab	0.5 ab
SUNTOP-205		17.67 ab	17.03 a	5.03 a	9.2 abcde	14.23 abcd	11.84 a	18.34 abc	0.42 ab	0.5 ab

SUNTOP-294	13.94 ab	16.41 a	4.2 ab	8.29 abcde	12.5 abcde	10.8 a	17.68 abc	0.38 ab	0.47 ab
SUNTOP-374	15.09 ab	15.92 a	2.9 ab	7.52 abcde	10.42 abcde	9.66 ab	16.96 abc	0.3 ab	0.44 ab
SUNTOP-439	10.56 ab	13.14 a	3.13 ab	6.57 bcde	9.7 bcde	9.32 ab	15.7 bc	0.33 ab	0.42 ab
SUNTOP-447	12.67 ab	16.02 a	4.29 ab	12.2 a	16.49 a	12.1 a	21.7 a	0.37 ab	0.57 ab
SUNTOP-65	13.56 ab	20.35 a	4.09 ab	8.14 abcde	12.22 abcde	10.38 ab	17.98 abc	0.4 ab	0.45 ab
Wallup	14.31 ab	14.35 a	3.83 ab	6.77 bcde	10.6 abcde	9.4 ab	16.1 abc	0.4 ab	0.42 ab
Westonia	17.24 ab	18.08 a	3.39 ab	6.95 bcde	10.34 abcde	10.22 ab	16.27 abc	0.33 ab	0.43 ab
UQBL2	15.56 ab	15.22 a	3.76 ab	8.09 abcde	11.85 abcde	9.72 ab	15.68 bc	0.38 ab	0.52 ab
Wylie	12.46 ab	13.6 a	1.98 b	4.93 de	6.92 e	6.75 b	13.2 bc	0.28 b	0.37 b
Yitpi	15.16 ab	13.2 a	4.27 ab	8.19 abcde	12.46 abcde	10.6 ab	16.98 abc	0.4 ab	0.48 ab
ZWB10-50	11.41 ab	12.78 a	4.71 a	9.63 abc	14.34 abc	11.23 a	18.48 ab	0.42 ab	0.52 ab

2 Supplementary Figure

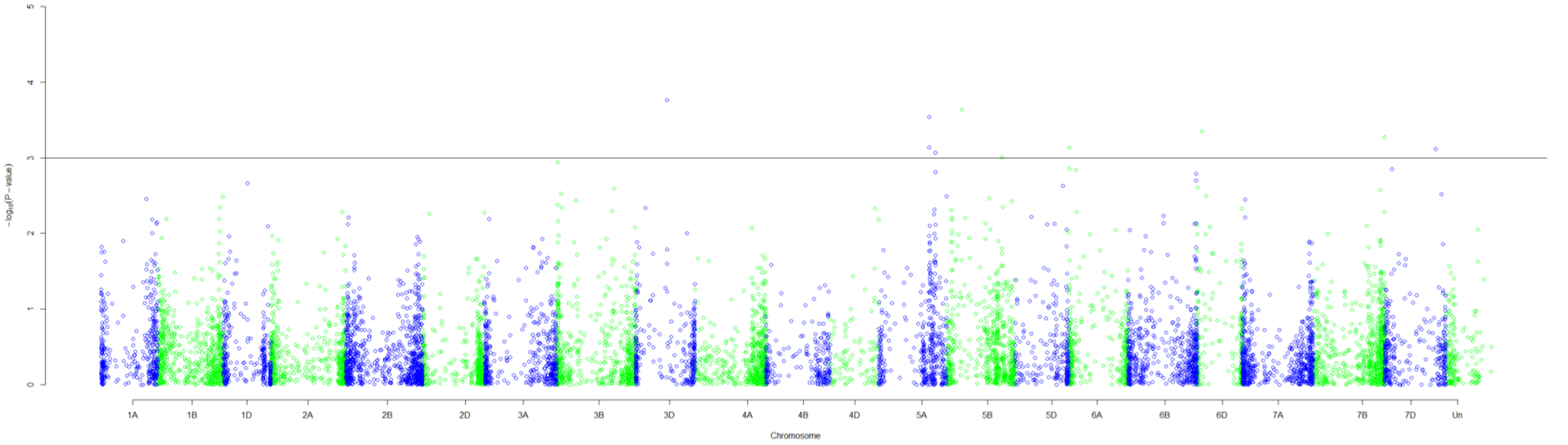

Supplementary Figure 1: Manhattan plot for NDVI15 at 29DAS. The horizontal line at log10 (***P***) = 3 indicates the chosen probability threshold to indicate a significant association with the trait.

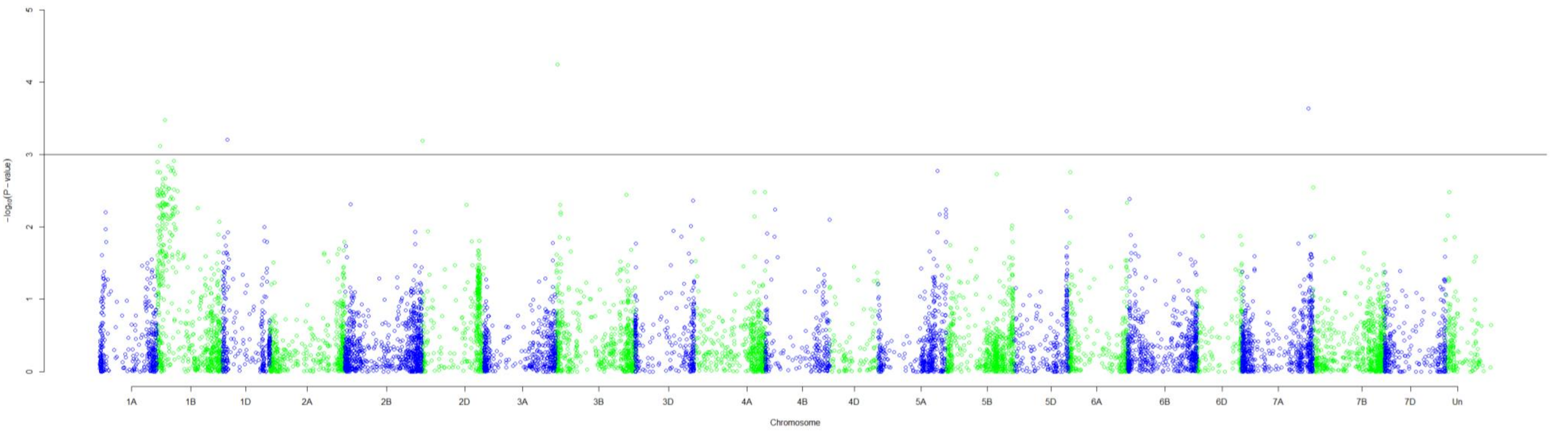

Supplementary Figure 2: Manhattan plot for NDVI16 at 21DAS. The horizontal line at log10 (***P***) = 3 indicates the chosen probability threshold to indicate a significant association with the trait.

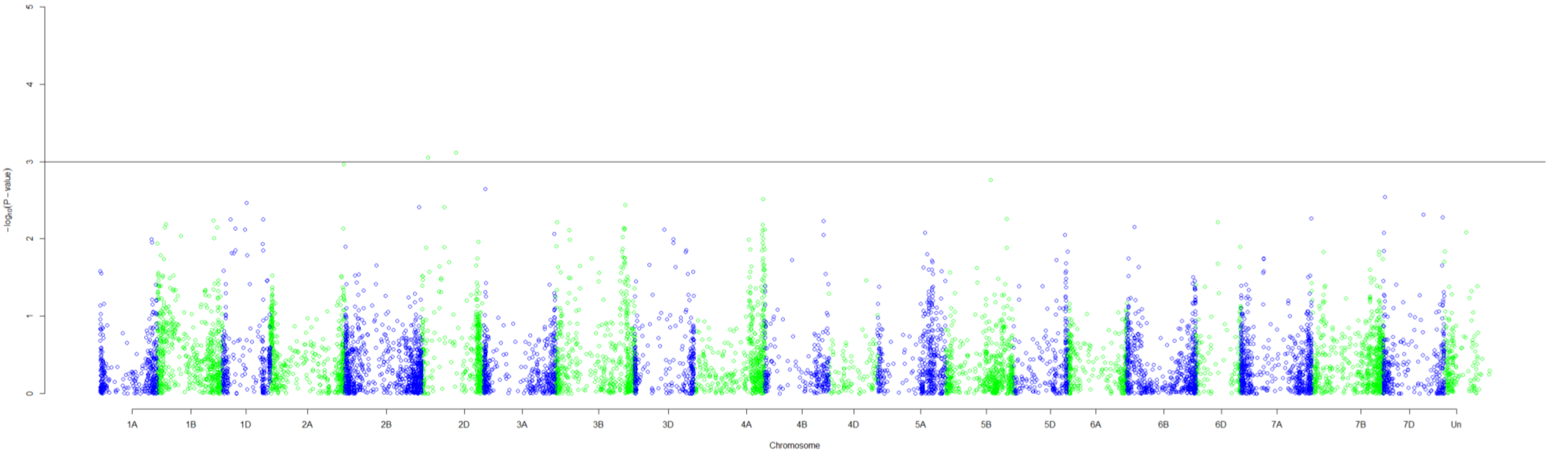

Supplementary Figure 3: Manhattan plot for NDVI17 at 29DAS. The horizontal line at log10 (***P***) = 3 indicates the chosen probability threshold to indicate a significant association with the trait.
